# Reassessing the environmental context of the Aitape Skull – The oldest tsunami victim in the world?

**DOI:** 10.1371/journal.pone.0185248

**Published:** 2017-10-25

**Authors:** James Goff, Mark Golitko, Ethan Cochrane, Darren Curnoe, Shaun Williams, John Terrell

**Affiliations:** 1 PANGEA Research Centre, School of Biological, Earth and Environmental Sciences, University of New South Wales, Sydney, New South Wales, Australia; 2 CNRS, Laboratoire Chrono-Environnement, Université de Bourgogne-Franche-Comté, Besançon, France; 3 Department of Anthropology, University of Notre Dame, Notre Dame, Indiana, United States of America; 4 Anthropology, School of Social Sciences, University of Auckland, Auckland, New Zealand; 5 ARC Centre of Excellence for Australian Biodiversity and Heritage and PANGEA Research Centre, School of Biological, Earth and Environmental Sciences, University of New South Wales, Sydney, New South Wales, Australia; 6 National Institute of Water & Atmospheric Research, Christchurch, New Zealand; 7 Integrative Research Center, Social Science, The Field Museum of Natural History, Chicago, Illinois, United States of America; University of Otago, NEW ZEALAND

## Abstract

There is increasing recognition of the long-lasting effects of tsunamis on human populations. This is particularly notable along tectonically active coastlines with repeated inundations occurring over thousands of years. Given the often high death tolls reported from historical events though it is remarkable that so few human skeletal remains have been found in the numerous palaeotsunami deposits studied to date. The 1929 discovery of the Aitape Skull in northern Papua New Guinea and its inferred late Pleistocene age played an important role in discussions about the origins of humans in Australasia for over 25 years until it was more reliably radiocarbon dated to around 6000 years old. However, no similar attention has been given to reassessing the deposit in which it was found—a coastal mangrove swamp inundated by water from a shallow sea. With the benefit of knowledge gained from studies of the 1998 tsunami in the same area, we conclude that the skull was laid down in a tsunami deposit and as such may represent the oldest known tsunami victim in the world. These findings raise the question of whether other coastal archaeological sites with human skeletal remains would benefit from a re-assessment of their geological context.

## Introduction

Over the past two decades we have become all too familiar with the devastating effects of large tsunamis on coastal populations and communities [1, 2], with the most notable being the 2004 Indian Ocean (2004 IOT) and 2011 Japan (2011 J) events, responsible for around 230,000 and 16,000 casualties respectively [1, 3]. However, there is nothing new in such tragedies. The history and prehistory of the Pacific region is punctuated by catastrophic tsunamis that have caused death, abandonment of coastal settlements, movement of people both inland and uphill, widespread loss of coastal resources, onset of warfare, breakdown of trading routes, and a rich record of oral traditions [4–8]. Geological and biological (micro- and macro-fossils) evidence has proved invaluable for improving our understanding of these past tsunamis throughout the region [9]. Interestingly though, while often reported in immediate post-tsunami surveys, vertebrate remains are rare in older deposits [10, 11], and there are few if any reliable examples of human skeletal remains [12]. This is somewhat surprising given the recognised catastrophic impact these events have had on human populations [8, 11]. Indeed, experience from recent events shows us that while in most cases bodies are removed for burial, there are inevitably many that are simply “missing” [13].

Here we report on the geological context for the Aitape Skull, which was originally discovered during a geological survey of northern PNG in 1929 at Paniri Creek, a location along the foothills of the Torricelli Mountains some 12 km inland from Sissano Lagoon, where a major tsunami struck in 1998 resulting in the deaths of more than 2000 people [14]. The site at Paniri Creek was revisited in 1962 [15] and later by us in 2014. We determine that the sediment in which this skull was recovered was deposited during a mid-Holocene palaeotsunami. The skull may be that of a tsunami victim, a signal of the increasing risk exposure of human populations as they increasingly settled coastal areas of the Pacific during the mid-Holocene.

### Prehistoric human occupation of northern PNG

There is currently little known archaeological evidence for significant settlement on the north coast of Papua New Guinea prior to about 2000 years BP, although people have occupied the region for at least the past 35,000 years [16]. Prior to the mid-Holocene, northern PNG’s steep coastal gradient likely ensured that the rocky coastline comprised of the Bewani-Torricelli-Barida ranges was relatively impoverished in subsistence resources and presented a significant barrier to intensive Pleistocene use of the coastline. Recorded archaeological sites of this period are primarily located in upland rockshelters [16–19].

Rapid coastal progradation following the stabilisation of sea level around 6000–7000 years ago started to create widespread coastal habitats favourable to human subsistence [20]. In the lower Sepik-Ramu basin (~230 km east of Paniri Creek) there is evidence of human exploitation of these new estuarine resources around 5,800 years BP [21, 22]. Evidence from the Aitape trough area indicates that these mid-Holocene estuarine environments were around 12–14 km inland from the present day coast in places (15). While there were most likely few prehistoric coastal communities in the region, it is reasonable to assume that they would have taken advantage of the newly forming lagoons and flat and may have relocated into these areas [20]. In settling the area, communities became exposed to living within a tectonically active environment and appear to have rapidly developed wide ranging social links [22, 23] that may have facilitated survival following natural disasters [20]. Indeed, it has only recently been recognised in the archaeological community that plate tectonics, geomorphology, and environmental change have likely had a powerful role to play in guiding human settlement and culture in northern PNG [20, 24]. Precursor events to the 1998 PNG tsunami undoubtedly left their mark.

### Paniri Creek and the Aitape Skull

**Fig 1:**
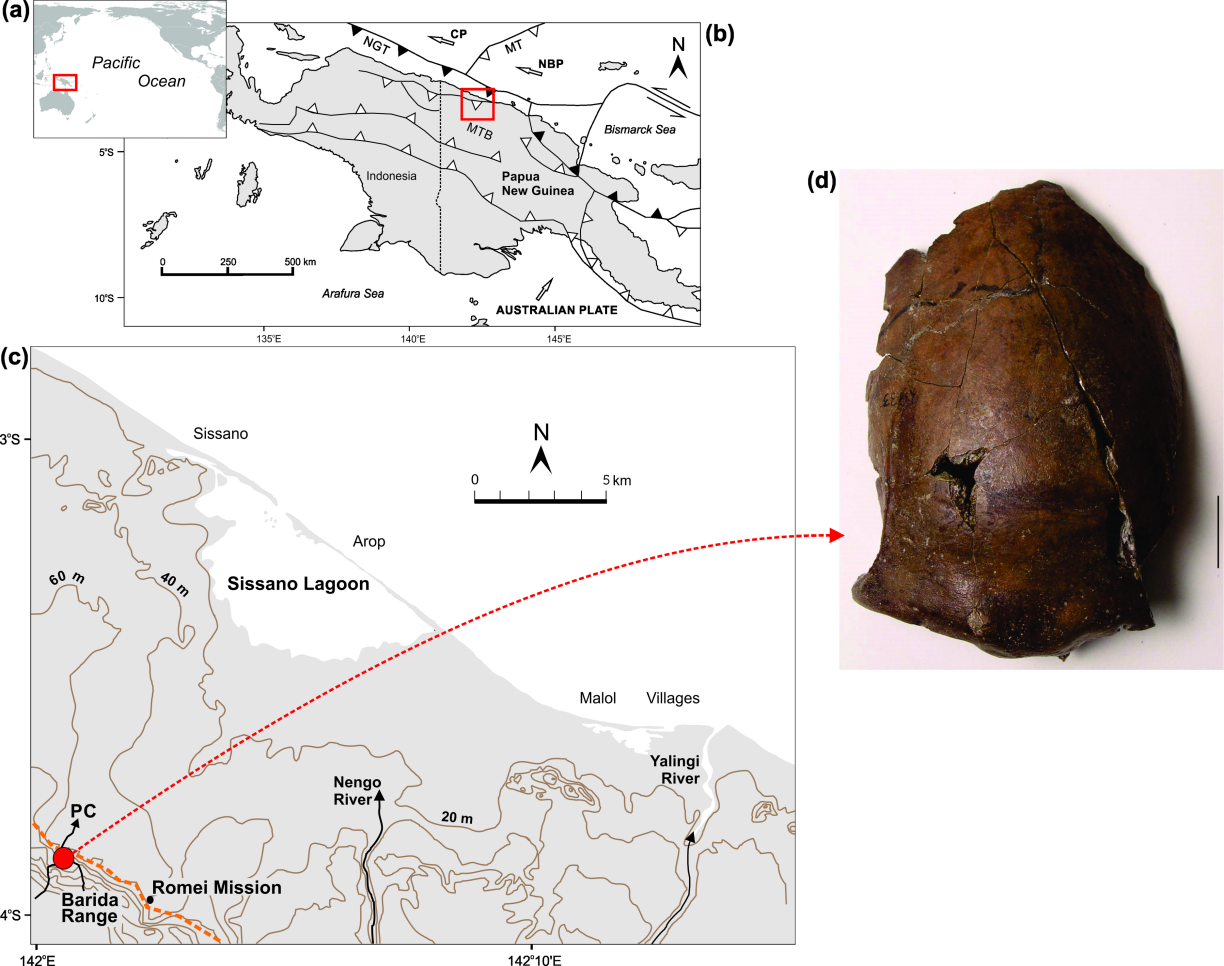
Site location and skeletal remains. (a) Location of New Guinea in the Pacific Ocean; (b) General study area in northern PNG (red square–see Fig 1C) with tectonic setting [26], CP: Caroline plate, MT: Melanesian trench, MTB: Mamberamo thrust belt, NBP: New Bismarck plate, NGT: New Guinea trench (arrows show approx. direction of plate movement); (c) Site of Aitape Skull where Paniri Creek exits the Barida Range approx. 11 km inland from Sissano Lagoon. Dashed orange line marks approx. edge of approx. 6000–7000 yr. old coastline [20]; (d) Aitape cranium: The early Holocene Aitape frontal bone (scale bar at lower right is 2 cm).

The Aitape Skull was found in the bank of Paniri Creek, about 12 km inland at an elevation of around 170 feet (~52 m) [15, 25](Fig 1). The site is immediately inland from Sissano Lagoon on the edge of the Barida Range that has uplifted ~52 m over the past 6000–7000 years (~1.00 mm/yr)(Fig 1).

**Fig 2:**
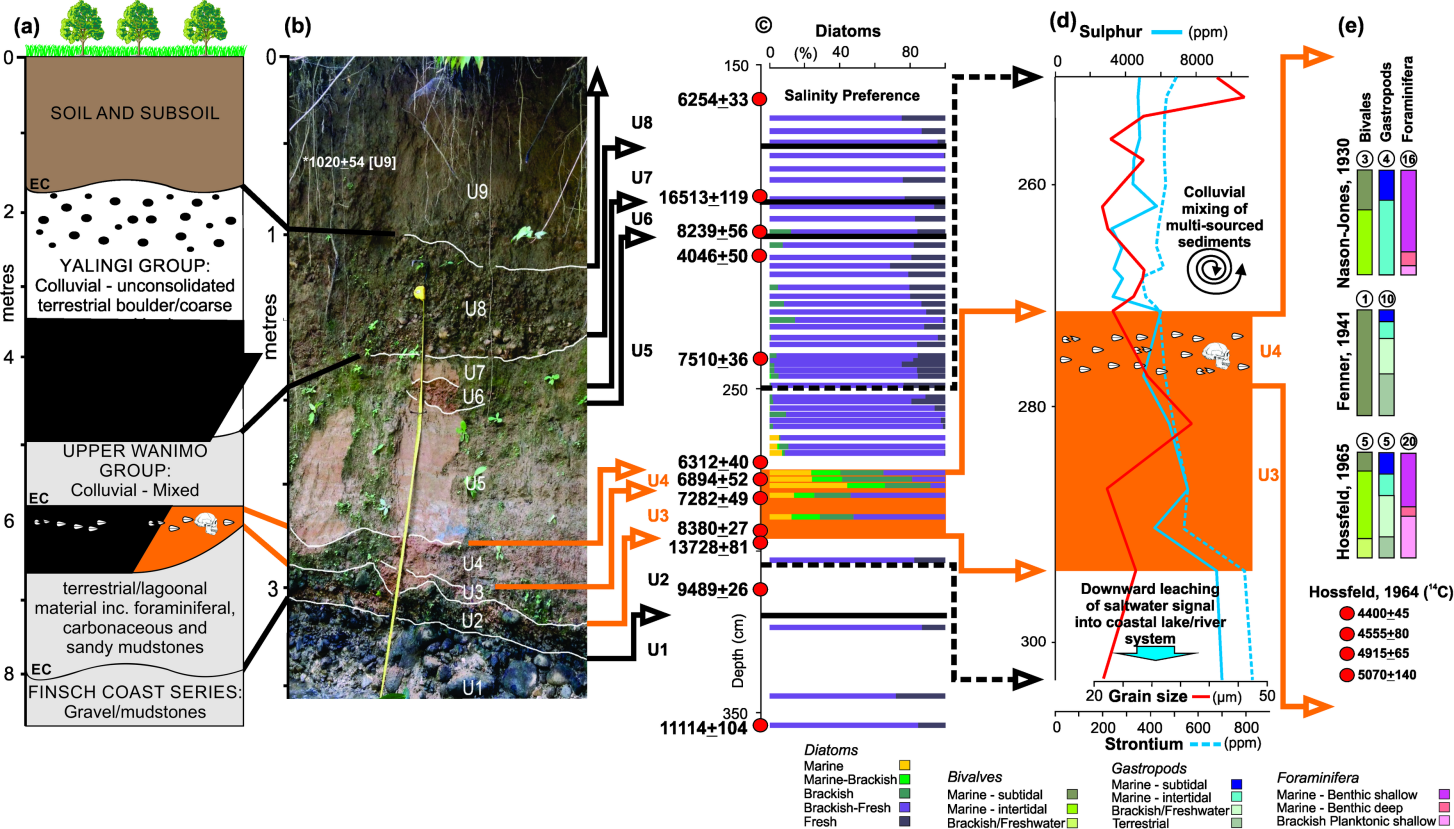
Paniri Creek study. (a) Stratigraphy of Paniri Creek section showing the Fossiliferous Lenticle (FL) containing the fragments of the Aitape Skull [15, 27, 28]; (b) Paniri Creek bank exposure stratigraphically correlated with earlier work [29], Units 3 and 4 correspond to Fossiliferous Lenticle and are highlighted by orange text and lines; (c) Diatom salinity preferences and radiocarbon dates for each unit (black solid arrows from 2b and horizontal black lines define contacts between units; red dots mark depth of ^14^C samples; dashed black arrows define upper and lower limits for 2d; orange shading and arrows define the lenticle; (d) Elemental concentrations for Sulphur and Strontium with the shell-rich component of the lenticle containing skull fragments shown within orange shaded area; (e) Macro-/micro-fossil and radiocarbon data for the lenticle recorded from past field studies in 1929 and 1962; circled numbers indicate number of species identified in each category reported by respective authors) (Supporting information is provided in S1–S6 Tables).

Stratigraphically, the skull fragments were within a scoured hollow of an intertidal mudflat. The sediment-infilled scour was termed a “Fossiliferous Lenticle” (hereafter referred to as "the lenticle"), and comprised carbonaceous sandy mud interbedded with coconut shell and fibre, driftwood, other plant remains, marine, intertidal and terrestrial shells and foraminifera [15, 27] (Fig 2). The lenticle was overlain by further intertidal mudflat sediments, about four metres of colluvial material of mixed origin, and soil (Fig 2A).

Skull fragments were collected from the lenticle in 1929. It has proven to be of remarkable palaeontological and archaeological interest. Indeed, it is rather striking that the Aitape Skull continues to be one of only two examples (the Watinglo mandible from near Vanimo is dated to *c*.10,000 years BP [30]) of human remains recovered from any site earlier than the later Holocene despite more than a century of work in PNG [28, 31, 32] (S1 and S2 Tables).

The skull is incomplete and comprises four major vault fragments: sections of the frontal including the nasal process, left and right parietals, and probable parts of the left sphenoid [23]. The initial description [23] continues to be the most detailed study of its morphology and affinities. It was concluded that the cranium probably derived from a 45 year old female and was morphologically most similar to recent Indigenous Australians particularly from the south of the continent. The lenticle was originally inferred to be of Lower Pleistocene age [14] and as such the Aitape Skull played an important role in discussions about the origins of humans in Australasia for the next 25 years [33, 34]. However, its importance diminished significantly once radiocarbon dating of various materials from the locality suggested it was mid-Holocene in age and that its antiquity had initially been greatly exaggerated [15, 35] (Fig 2E).

Subsequent work [36] has questioned the earlier sex diagnosis [28], proposing instead that the Aitape Skull remains might be from a male. Regarding its affinities, it was suggested that the cranium is lightly built (as is the Watinglo mandible) and shows affinities to recent New Guineans [37]. More recently, however, a metrical study of the partial calvaria tentatively concluded that it most closely resembled Pleistocene-early Holocene crania from Southeast Australia rather than recent New Guineans or Indigenous Australians [38].

### Tsunamis and geomorphological change on a tectonically active coastline

The northern coast of PNG has a history of at least seven locally significant tsunamis since 1907 [39], with the 17 July 1998 event being the most well-known along this tectonically active coastline. In 1998, waves of up to 15 m high (flow depth) inundated the Sissano Lagoon (hereafter referred to as "the lagoon") area (Fig 1) and penetrated up to 5 km inland [40]. Initially, tsunamigenesis was considered to be by fault rupture with or without co-seismic subsidence [40, 41], but subsequently a Submarine Mass Failure (SMF) origin was proven [42].

Local tsunamigenesis in the lagoon area is related to its position adjacent to the New Guinea trench, an active subduction zone on the leading edge of the Australian tectonic plate, which is in a geologically rapid oblique collision with a number of micro-plates in the Bismarck Sea [43] (Fig 1B). In particular, convergence between the North Bismarck and Australian plates is ~70 mm/year with lateral shear of ~100 mm/year. Not surprisingly, large earthquakes occur frequently in the zone of deformation between these plates, with rapid uplift of the Barida Range (~1.0 mm/yr) and subsidence of the lagoon in the seaward Aitape trough (Fig 1C).

Prior to co-seismic subsidence of 1.8–3.6 m during the 1907 earthquake, the lagoon was a coastal lake [44]–the now semi-enclosing spit was a complete barrier at that time. Indeed, the shallow lagoon (~1.0 m deep) can be seen as a transient geomorphological feature representing a balance between high sediment supply and co-seismic subsidence. High volumes of sediment input from the Yalangi and Bliri (7 km NW of the lagoon) Rivers produce rapidly prograding deltas, maintain and enhance the lagoonal spit/barrier, and introduce large quantities of unconsolidated material to the narrow inner trench slopes that reach 3500 m deep about 30 km offshore [40]. Co-seismic subsidence of around 40–70 cm was noted in 1998 [40, 45], reducing the surface expression of the enclosing spit. However, it is not only the interplay between sediment supply to the spit/barrier and co-seismic subsidence that drives this process. Cores taken following the 1998 PNG tsunami indicate that the main sediment supply infilling the lagoon was from large tsunamis reworking much of the spit and nearshore sediments during inundation [40].

## Materials and methods

### Ethics statement

This study was carried out on private land. Permission to conduct archaeological and geological survey work was obtained from the Papua New Guinea National Research Institute, the Papua New Guinea National Museum and Art Gallery, the Sandaun provincial government (Vanimo), the Aitape-Lumi district administration, and the Aitape-Lumi West LLG manager (John Akove). Permission to collect samples was obtained from John Sairi, the owner of the land on which the Paniri Creek site is located. Field studies did not involve endangered or protected species.

No new fossil or archaeological specimens were collected as part of this study and the work is based upon evidence reported from earlier publications. Geological material only was collected during this study and all relevant material is held at the Field Museum of Natural History in Chicago.

### Paniri Creek studies

In 1929, stratigraphy was interpreted in the field. Macrofossil samples including the Aitape Skull were taken for visual identification in the laboratory. Foraminifera were processed by “floatings” with no further details given [14, 27, 28] (S3 and S4 Tables). Macrofossils and foraminifera sampled in 1962 were processed by the same methods [15] (S5 Table).

In 2014, samples were taken at around 2 cm intervals throughout the streambank section at the immediate juncture of the Kiyen and Kangkonggalle Creeks where they flow into Paniri Creek, estimated to be within 200–300 meters of the original skull site [15] (Fig 1, p. 162: “*The soft fossiliferous mudstone that contained the human remains outcrops in most of the creeks where they leave the hills and enter the plains*”). Organic matter content (LOI) was determined on a dry weight basis by ashing at 550°C for 4 h [46]. Samples for grain size analysis were first treated with hydrogen peroxide to remove organic matter and then analysed by laser diffraction using a Malvern Mastersizer 2000. A suite of grain size parameters were calculated using GRADISTAT software [47], including percentages of sand, silt and clay, graphic mean, skewness and kurtosis [48]. LOI and grain size data were analysed specifically to help determine that nature of deposition, a useful indicator in helping to differentiate between storm and tsunami sediments [5].

Semi-quantitative geochemical analysis was conducted using a Niton Goldd+ handheld XRF unit. Samples were dried and then finely powdered using an agate mortar and pestle. For each sample, 5 gm of the resulting powder was weighed into an XRF sample cup, firmly tamped down and measured twice using the instrument’s “Mining Cu/Zn” mode for a total of 110 seconds per sample (varying beam energy and voltage to measure low, medium, and high mass elements). The resulting averaged fundamental parameters values were then calibrated against a set of 12 USGS and NIST certified powdered rock and sediment standards as well as New Ohio Red Clay, a powdered commercial clay widely utilized in archaeometric studies with well measured concentrations. Two powdered standards (NIST679 ‘Brick Clay’ and USGS SDC-1 ‘mica-schist’) were run with each batch of samples to monitor instrument performance. 21 elements were consistently measured in the samples (Al, Si, P, S, K, Ca, Ti, V, Cr, Mn, Fe, Co, Ni, Cu, Zn, Rb, Sr, Y, Zr, Nb, and Pb; with relevant S and Sr data produced in Fig 2). When used in conjunction with other analyses, variations in elemental geochemistry can be a key indicator of not only marine, but more specifically, tsunami inundation [5].

Diatom samples were prepared following standard methods [49]. The identification of species was based on standard diatom floras [50–52]. Diatom assemblages in coastal sediments vary depending upon the depositional process involved. These data coupled with sedimentary and geochemical evidence help to better identify the nature of such events.

AMS dating was conducted at the University of Georgia Center for Applied Isotope Studies (CAIS). Dates were calibrated in OxCal v. 4.2 using the SHInt13 Southern Hemisphere calibration curve (Fig 2C). Little if any macroscopic organic material was identified in the Paniri Creek samples, with the exception of a handful of small shell and charcoal fragments embedded directly in the face of unit 4, profile 1. These materials returned recent dates that likely reflect embedding during flooding of the stream bed and as such have not been included in the main text. All other C-14 measurements reflect bulk carbonate extraction from sediment (S1 Table). Four earlier radiocarbon samples collected in 1962 were analysed at DSIR Institutes of Nuclear Sciences, New Zealand (three) and Gakushuin University, Japan (one). No further details are provided [15, 25] (S2 Table).

### Paniri Creek and the Aitape Skull—Analysis and interpretation

Three sets of micro- and macrofossil assemblages were collected and examined by original team members, two in 1929 and one on a later visit in 1962 [14, 15, 28] (Fig 2E; also refer to SI for details of all gathered material). Macrofossils and vegetation were typically of Indo-Pacific lagoonal origin, with none of the common shallow water Indo-Pacific foraminifera present. The remaining foraminiferal species were mostly characteristic of nearshore waters with one from the deeper continental shelf. The interpretation was of deposition in a coastal mangrove swamp inundated by water from a shallow sea [15].

Our recent resampling from near the original skull find site [29], has provided an opportunity to better understand the depositional environment and context for the lenticle through grain size, geochemistry and diatom analyses (Fig 2; S3 Table). This finer resolution study extends that of earlier work [15]. The lenticle represents a higher energy, markedly marine incursion into an alternating coastal lake/river system with little or no saltwater influence (similar to the pre-1907 lagoon). This incursion precipitated an environmental change leading to a more open lagoonal system. The change was most likely caused by erosion of the coastal barrier as noted in more recent events [40]. Subsequent colluvial activity and uplift gradually isolated the site from the sea (Fig 2). Of particular note within the lenticle is a multi-proxy record including sediments and a diatom assemblage remarkably similar to those of the 1998 PNG tsunami [32, 40, 53], a geochemical signature indicative of downward leaching of saltwater elements (S, Sr) into the underlying sediments [54], and a record of deep benthic foraminifera (Fig 2). This unique combination of shallow- and deep-water sediments and intact microfossils points to a tsunami as opposed to a storm origin [5]. As a tsunami moves through the deep ocean, it can disturb and entrain material from as much as 1-km depth [55], well below any storm wave base. As such the multi-proxy evidence presented here is indicative of sediments laid down by a tsunami [5].

### The oldest tsunami victim in the world?

The ultimate interpretation of the general depositional setting for the Aitape Skull from earlier studies (using primarily bivalve, gastropod and foraminiferal data; S3–S5 Tables) was that of a coastal mangrove swamp, probably exposed at low tide and inundated at intervals by water from a shallow muddy protected sea [15]. More specifically, the skull was found in “a lenticle representing sedimentation in a scour in a mangrove swamp”, with an age of around 5335–6180 years BP [15, 25] (S2 Table). A recent re-analysis of the original data, coupled with additional material from grain size, diatom, geochemical and radiocarbon data (S1 and S6 Tables) has highlighted the unique nature of the lenticle in that it represents a notably high energy marine incursion into an essentially freshwater coastal lake as opposed to lagoon. Further radiocarbon dating places the age around 6300–7000 years BP.

Comparison with the known geomorphological history of Sissano Lagoon suggests that initial post mid-Holocene progradation of northern PNG occurred across the edge of the Aitape trough and this undoubtedly affected the coastal lake/lagoonal environment of the Aitape Skull. Indeed, the transformation from lake to lagoon around 6000 years ago mirrors that reported for the lagoon in 1907. We were unable to determine any subsidence associated with the skull setting, but the unique introduction of marine sediments that encased the Aitape Skull is consistent with conditions reported following the 1998 PNG tsunami [40, 53]. Subsequent environmental conditions diverge from those experienced on the Aitape trough, with rapid ongoing uplift starting possibly immediately during (co-seismic) or sometime after inundation.

We seek to modify the original interpretation [15] that the site was “probably exposed at low tide and inundated at intervals by water from a shallow muddy protected sea” to “a coastal brackish/freshwater lake inundated on at least one occasion by a high-energy marine incursion across a shallow sea”. Given the active tectonics of the region and the historical record of fault rupture/SMF tsunamigenesis [40, 42, 45], and similarities with evidence from the 1998 PNG event [40, 53, 56] we consider that the marine incursion represents inundation by a tsunami around 6000–6500 years ago.

Our reinterpretation of the environmental context at Paniri Creek thus warrants a reinterpretation of how the Aitape Skull arrived at its place of final deposition. Three possible mechanisms may be proposed. Firstly, the Aitape Skull could represent a victim killed during palaeotsunami inundation itself. While victims of recent tsunamis including the 2004 IOT have typically been recovered largely intact, and therefore would enter the archaeological record in an articulated state, there are reasons to believe that similar events on the north coast of PNG might produce scattered and disarticulated remains.

During the 1998 PNG event, the resulting tsunami wave was largely clean of sediment until it reached the beach and only had 200 m of low-lying spit to traverse before entering the lagoon [40]. It was consequently moving more rapidly than many other recent tsunamis (~40 mph at 200 m inland and probably faster at the beachfront [57, 58],–for similar distances inland, tsunami speeds have been: ~15 mph—2004 IOT in India and Thailand [59, 60], ~20 mph– 2011 J [61], ~10 mph– 2009 Samoa [62]), and at an unusually sustained speed that on land adjacent to the lagoon was still ~25 mph some 600 m inland [58]. Once on land, the wave was able to entrain a considerable quantity of sand, building debris and trees, causing death by sand blasting, dismemberment, impact and drowning [40, 63, 64].

In such environments, many bodies become buried in sediment sinks (the lagoon) and are unlikely to be retrieved [65]. Following the 1998 PNG event, attempts to retrieve victims from the lagoon were called off a week after the tsunami because crocodiles were feeding on the corpses, but dismembered bodies continued to be found in subsequent days [66, 67]. A similar set of events during the mid-Holocene could account for the Aitape Skull. It should be noted also that Hossfield spent only four hours at the Aitape Skull site, and notes that the search for further human remains was “not exhaustive” [27].

Secondly, past mortuary tradition in the Aitape area included such practices as dismemberment, curation of skulls after defleshing, and above ground ossuaries [68]. The skull may therefore represent the remains of a near-contemporaneous burial of an individual previously buried or exposed on the incipient coastal flats north of the Paniri Creek site. However, it is notable that following the 1998 PNG event, one of the authors (JG) observed that bodies buried in a modern cemetery were not entrained even though all the sediment above them had been removed by the tsunami.

A third alternative is that the skull represents either a more recently emplaced individual (i.e., within the last few hundred years), or a much older burial that was washed out during the Paniri tsunami event. We hold both of these possibilities to be unlikely. While units 3–4 in our 2014 profile did produce two relatively recent dates on organic material from near the profile face (see supplemental material), these units are bracketed by a consistent set of dates placing this overall part of the 2014 Paniri sequence firmly in the mid-Holocene. As the deposit is currently 12 km inland, it is unlikely that the tsunami represented in these units is of later age. Given the rapid rate of deposition evident in the Paniri sequence, if the individual in question had been buried far earlier than the tsunami event preserved at Paniri Creek, it is less likely that they would have been washed out during the event.

Therefore, on balance, we would argue that the individual in question was either directly killed in the mid-Holocene tsunami, or redeposited from a burial dating to slightly earlier than the Paniri event. Given that the skull is not fossilized [38], it might be possible to directly radiocarbon date it in the future to demonstrate or refute a chronological assignment to the mid-Holocene, assuming diagenetic effects [69] can be accounted for.

It seems reasonable to suggest that as people in the SW Pacific began to occupy coastal environments during the mid-Holocene, they would have been increasingly impacted by environmental risks including tsunamis like the one we have documented at Paniri Creek. Coupled with other risk factors such as intensification of the El Niño/Southern Oscillation (ENSO) cycle that also occurred during this time [70, 71, 72], tsunamis may have contributed to a much more dynamic world of community and individual mobility and an increasing reliance on risk-mitigation strategies including the fostering and maintenance of wider-ranging social ties, and therefore likely played a significant role in the spread of materials and new ideas and practices throughout the SW Pacific as documented in the mid-Holocene archaeological record.

While it may never be possible to definitively assign the Aitape Skull as the earliest tsunami victim in the world, this reassessment of the existing work indicates that the Sissano Lagoon region may well contain an extensive Holocene record of human interactions with catastrophic events such as tsunamis. Further research is therefore needed to determine that nature and extent of these interactions.

## Supporting information

S1 Table Radiocarbon data from 2014 study. Samples within the grey shaded area were discounted from this study since they are most likely contaminated by recent carbon at or near the surface or the profile.
(DOCX)

S2 Table Radiocarbon data from 1962 study. Materials were obtained from “*within the fossiliferous lenticle*” [15, 16]. No further sample details were provided—CRA is inferred to be at 1σ. No CAR was provided other than what we inferred to be a calibrated mid-point (2450 BC, 2605 BC, 2965 BC and 3120 BC respectively). The close agreement between the ages of the two carbonised wood samples was considered to better approximate the time of the skull’s deposition [15, 16]. OxCal 4.2 was used to provide the CARs in the table. n.b. Tests on the uranium content of the skull by Dr. K. Oakley (British Museum) determined a late Pleistocene to Recent age [15].
(DOCX)

S3 Table First collection. (DOCX)

S4 Table Second collection. Additional material.
(DOCX)

S5 Table Third collection. (DOCX)

S6 Table Diatom taxa identified in 2014 study. The affinity and lifeform is given after each taxon (Affinitiy: P—Polyhalobous [marine], M–Mesohalobous [marine-brackish], OH—Oligohalobion halophilous [brackish-fresh], OI—Oligohalobion indifferent [fresh-brackish], H–Halophobous [fresh]; Lifeform, the second (or third) letter: P—Planktonic/tychoplanktonic, E–Epiphytic [inc. species classified as epontic], B–Benthic).
(DOCX)
