# Supplementary material for: Reassessing the environmental context of the Aitape Skull – The oldest tsunami victim in the world?
Source: PLoS One. 2017 Oct 25;12(10):e0185248. doi: 10.1371/journal.pone.0185248 (PMC5656299; doi:10.1371/journal.pone.0185248)
Supplement: S1 Table — Samples within the grey shaded area were discounted from this study since they are most likely contaminated by recent carbon at or near the surface or the profile. (DOCX) [file pone.0185248.s001.docx]

**Radiocarbon data**

| Laboratory Code | Unit | | Depth (cm) |  | Material | CRA (BP 1σ) | % Modern Carbon  (1σ) | δ^13^‰ | CAR (cal BP, 95.4%)* | Mean Calibrated Age (cal BP, 95.4%) |  |  |
| --- | --- | --- | --- | --- | --- | --- | --- | --- | --- | --- | --- | --- |
| UGAMS20409 | | 1 | 354 |  | soil bulk carbonate | 9750 ± 40 | 29.71 ± 0.15 | -26.8 | 11234-10824 | 11114± 104 |  |  |
| UGAMS20410 | | 2 | 314 |  | soil bulk carbonate | 8510 ± 30 | 34.68 ± 0.12 | -25.1 | 9532-9441 | 9489 ± 26 |  |  |
| UGAMS20411 | | 2 | 294 |  | soil bulk carbonate | 11960 ± 40 | 22.57 ± 0.12 | -24.7 | 13951-13565 | 13728 ± 81 |  |  |
| UGAMS26972 | | 3 | 284-294 |  | soil bulk carbonate | 7610 ± 30 | 38.76 ± 0.13 | -27.2 | 8424-8330 | 8380 ± 27 |  |  |
| UGAMS20412 | | 3 | 279-284 |  | soil bulk carbonate | 6390 ± 25 | 45.14 ± 0.14 | -26 | 7414-7173 | 7282 ± 49 |  |  |
| UGAMS26971 | | 4 | 274-277 |  | soil bulk carbonate | 6090 ± 25 | 46.86 ± 0.15 | -26.9 | 6983-6794 | 6894 ± 52 |  |  |
| UGAMS20054 | | 4 | 274-277 |  | charcoal from profile surface; charcoal, | 750 ± 20 | 91.13 ± 0.24 | -28.6 | 680-650 | 650 ± 31 |  |  |
| UGAMS20420 | | 4 | c. 275 |  | aggregated soil, and shell fragments from near profile surface | 2450 ± 20 | 73.66 ± 0.2 | -25.8 | 2684-2349 | 2453 ± 91 |  |  |
| UGAMS26970 | | 5 | 272 |  | soil bulk carbonate | 5550 ± 25 | 50.13 ± 0.16 | -27 | 6398-6218 | 6312± 40 |  |  |
| UGAMS20413 | | 5 | 242 |  | soil bulk carbonate | 6660 ± 25 | 43.66 ± 0.14 | -23 | 7569-7440 | 7510 ± 36 |  |  |
| UGAMS20414 | | 5 | 208 |  | soil bulk carbonate | 3750 ± 20 | 62.69 ± 0.18 | -26.4 | 4149-3934 | 4046 ± 50 |  |  |
| UGAMS20415 | | 6 | 202 |  | soil bulk carbonate | 7440 ± 30 | 39.6 ± 0.13 | -25.2 | 8341-8073 | 8239 ± 56 |  |  |
| UGAMS20416 | | 7 | 188 |  | soil bulk carbonate | 13730 ± 40 | 18.1 ± 0.1 | -24.2 | 16765-16295 | 16513 ± 119 |  |  |
| UGAMS20417 | | 8 | 159-164 |  | soil bulk carbonate | 5500 ± 25 | 50.44 ± 0.15 | -25.2 | 6306-6201 | 6254 ± 33 |  |  |

*SHcal13 Calibrated using OxCal 4.2

**S1 Table. Radiocarbon data from 2014 study.** Samples within the grey shaded area were discounted from this study since they are most likely contaminated by recent carbon at or near the surface or the profile.
