# Supplementary material for: Reassessing the environmental context of the Aitape Skull – The oldest tsunami victim in the world?
Source: PLoS One. 2017 Oct 25;12(10):e0185248. doi: 10.1371/journal.pone.0185248 (PMC5656299; doi:10.1371/journal.pone.0185248)
Supplement: S2 Table — Materials were obtained from “within the fossiliferous lenticle” [15, 16]. No further sample details were provided—CRA is inferred to be at 1σ. No CAR was provided other than what we inferred to be a calibrated mid-point (2450 BC, 2605 BC, 2965 BC and 3120 BC respectively). The close agreement between the ages of the two carbonised wood samples was considered to better approximate the time of the skull’s deposition [15, 16]. OxCal 4.2 was used to provide the CARs in the table. n.b. Tests on the uranium content of the skull by Dr. K. Oakley (British Museum) determined a late Pleistocene to Recent age [15]. (DOCX) [file pone.0185248.s002.docx]

| Laboratory Code | Material | | Inferred  CRA (BP 1σ) | CAR (cal BP, 95.4%)* | |  |
| --- | --- | --- | --- | --- | --- | --- |
| N.Z. R.1131/1 | Molluscs (*Melania*) | | 4400 ± 85 | 5298-4714 |  | |
| N.Z. R.1131/2 | Carbonised coconut shell | | 4555 ± 80 | 5446-4876 |  | |
| N.Z. R.1131/3 | Carbonised wood | | 4915 ± 65 | 5856-5335 |  | |
| GaK-440 | Carbonised wood | | 5070 ± 140 | 6179-5475 |  | |
|  | |  |  |  |  |  |

*SHcal13 Calibrated using OxCal 4.2

**S2 Table. Radiocarbon data from 1962 study.** Materials were obtained from “*within the fossiliferous lenticle*” [15, 16]. No further sample details were provided - CRA is inferred to be at 1σ. No CAR was provided other than what we inferred to be a calibrated mid-point (2450 BC, 2605 BC, 2965 BC and 3120 BC respectively). The close agreement between the ages of the two carbonised wood samples was considered to better approximate the time of the skull’s deposition [15, 16]. OxCal 4.2 was used to provide the CARs in the table. n.b. Tests on the uranium content of the skull by Dr. K. Oakley (British Museum) determined a late Pleistocene to Recent age [15].
