# Supplementary material for: Reassessing the environmental context of the Aitape Skull – The oldest tsunami victim in the world?
Source: PLoS One. 2017 Oct 25;12(10):e0185248. doi: 10.1371/journal.pone.0185248 (PMC5656299; doi:10.1371/journal.pone.0185248)
Supplement: S3 Table — (DOCX) [file pone.0185248.s003.docx]

**Macrofossils, Microfossils and other material collected and analysed**

1. **Nason-Jones [14]:**

In 1929 “palaeontological data” were recovered from the specimens examined. “*In addition to the foregoing, a carbonised coconut was found, together with a fragment of a human skull. A search for teeth and other relics was conducted without success*”.

Pelecypoda = Bivalvia (marine & freshwater molluscs)

*Arca nodosa*

*Paphia*

*Placenta mandirant janensis*

Gasteropeda - Gasteropoda

*Neritina sp.*

*Melania woodwardi*

*Melania denisoniensis*

*Melania scabra*

Foraminifera

*Cibicides praecinctus*

*Cristellaria orbicularis*

*Cristellaria cultrate*

*Cristellaria calcarata*

*Eponides tumidus*

*Eponides procera*

*Epistomena elegans*

*Globigerina triloba*

Heterostegina sp.

*Operculinella venosa*

*Operculina granulosa*

*Polystomella craticulata*

*Quinqueloculina lamarckiana*

Rotalia sp. nov

*Rotalia schroeteriana*

*Rotalia papillosa*

**S3 Table. First collection**
