# Supplementary material for: Reassessing the environmental context of the Aitape Skull – The oldest tsunami victim in the world?
Source: PLoS One. 2017 Oct 25;12(10):e0185248. doi: 10.1371/journal.pone.0185248 (PMC5656299; doi:10.1371/journal.pone.0185248)
Supplement: S4 Table — Additional material. (DOCX) [file pone.0185248.s004.docx]

**Macrofossils, Microfossils and other material collected and analysed**

1. **Fenner [19]:**

Some additional macrofossils from the 1929 collection.

Pelecypoda = Bivalvia

*Arca (Tegillarca) granosa Linn.*

Gasteropeda - Gasteropoda

*Telescopium fuscum Schumacher*

Papuina sp (land shell)

*Neritina cornea Linn.*

*Neritina souverbiana Montrouzier*

Laoma sp.

Cyclophorus sp.

*Melania cf. juncea Lea*

*Melania cf. recta Lea*

*Melania cf. canaliculata Reeve*

*Cyrena coaxans Gmelin*

*Cocos nucifera* (Coconut palm)

**S4 Table. Second collection.** Additional material
