# Supplementary material for: Reassessing the environmental context of the Aitape Skull – The oldest tsunami victim in the world?
Source: PLoS One. 2017 Oct 25;12(10):e0185248. doi: 10.1371/journal.pone.0185248 (PMC5656299; doi:10.1371/journal.pone.0185248)
Supplement: S5 Table — (DOCX) [file pone.0185248.s005.docx]

**Macrofossils, Microfossils and other material collected and analysed**

1. **Hossfeld [15]:**

In 1962 when the site was revisited, “*Marine shells, carbonized coconut fibre, and shell and wood fragments were excavated from the soft blue mudstone, cleaned, sun-dried, then packed in clean 'polythene bags. The work was terminated by collapse of the bank*.” It was noted that “***The soft, fossiliferous mudstone that contained the human remains outcrops in most of the creeks where they leave the hills and enter the plain.*”**

Pelecypoda = Bivalvia (marine & freshwater molluscs)

*Arca (Tegillarca) granosa Linnaeus*

*Placuna placenta Linnaeus*

*Dosinia sculpta Hanley*

*Crassostrea* sp.

Cyrena coaxana Gmelin

Gasteropeda - Gasteropoda

*Telescopium telescopium Linnaeus*

*Melania juncea Lea*

*M. recta Lea*

*M. canaliculata Reeve*

*Neritina souverbiana Montrouzier*

Foraminifera

? *Psammosphaera fusca testacea* Flint

*Haplophragmoides* sp.

*Textularia* cf. *sagittula* Defrance

*Bulimina rostrata* Brady

*B. barbata* Cushman

*B. striata mexicana* Cushman

*Uvigerina* sp. *(pigmea* group)

*U. asperula* Czjek

U. sp.

*Siphogenerina virgula* (Brady)

*Bolivina* aff. *pulchella* d'Orbigny

*B.* sp.

*Globigerina eggeri* Phumbler

G. cf. *triloba* Reuss

*Orbulina universa* d'Orbigny

*Sphaeroidina bulloides* d'Orbigny

*Sphaeroidinella dehiscens* (Parker and Jones)

*Pullenia bulloides* d'Orbigny

*Globorotalia tumida* (Brady)

*Cibicides* aff. *cicatricicosus* (Schwager)

**S5 Table. Third collection**
