# Supplementary material for: Reassessing the environmental context of the Aitape Skull – The oldest tsunami victim in the world?
Source: PLoS One. 2017 Oct 25;12(10):e0185248. doi: 10.1371/journal.pone.0185248 (PMC5656299; doi:10.1371/journal.pone.0185248)
Supplement: S6 Table — The affinity and lifeform is given after each taxon (Affinitiy: P—Polyhalobous [marine], M–Mesohalobous [marine-brackish], OH—Oligohalobion halophilous [brackish-fresh], OI—Oligohalobion indifferent [fresh-brackish], H–Halophobous [fresh]; Lifeform, the second (or third) letter: P—Planktonic/tychoplanktonic, E–Epiphytic [inc. species classified as epontic], B–Benthic). (DOCX) [file pone.0185248.s006.docx]

| *Achnanthes affinis Grun. OIE* |
| --- |
| *Achnanthes hungarica Grun. OIE* |
| *Achnanthes lanceolata (Bréb.) Grun. OIE* |
| *Achnanthes minutissima Kütz. OIE* |
| *Amphora salina W. Smith OHB-MB* |
| *Amphora spp. OI-OH* |
| *Amphora veneta Kütz. OHB* |
| *Aulacoseira granulata (Ehr.) Simonsen OIP* |
| *Brachysira serians (Bréb.) Round & Mann OIB-HB* |
| *Brachysira styriaca (Grun.) Round & Mann OIB-HB* |
| *Cocconeis scutellum Ehr. PE* |
| *Cymbella affinis Kütz. OIE* |
| *Eunotia diodon Ehr. HE* |
| *Eunotia formica Ehr. OIE* |
| *Eunotia veneris (Kütz) De Toni OIE-HE* |
| *Fragilaria construens (Ehr.) Grun. OIE* |
| *Fragilaria construens var. venter (Ehr.) Grun. OIP* |
| *Frustulia magaliesmontana Cholnoky OIB* |
| *Frustulia rhomboides (Ehr.) De Toni HP* |
| *Gomphonema parvulum (Kütz.) Kütz. OIE* |
| *Gomphonema spp. OI* |
| *Gomphonema valentinica Nikolajev OIE* |
| *Hantzschia amphioxys (Ehr.) Grun. OIB* |
| *Melosira moniliformis PE\PP-MP\ME* |
| *Melosira westii W. Smith PP* |
| *Navicula aucklandica Grun. PB* |
| *Navicula auriculata Hust. OIE* |
| *Navicula clementis Grun. OHB-OIB* |
| *Navicula halophila (Grun.) Cleve. MB* |
| *Navicula ilopangoensis Hust. OIB* |
| *Navicula tripunctata (Müll.) Bory. OIB-HB* |
| *Nitzschia sigma (Kütz.) W. Smith MB* |
| *Paralia sulcata (Ehr.) Cleve. PP* |
| *Pinnularia graciloides Hust. OIB* |
| *Pinnularia stomatophora (Grun.) Cleve. OIB* |
| *Rhopalodia gibba (Ehr.) Müll. OIE* |
| *Stauroneis dubitabilis Hust. OIB* |
| *Stauroneis phoenicenteron (Nitzsch) Ehr. OIB* |
| *Surirella elegans OIB* |
| *Surirella ovata Kütz. OIB-OIE* |
| *Synedra investiens W. Smith PE* |
| *Tabellaria flocculosa (Roth.) Kütz. HE* |
| *Triceratium favus Ehr. PB* |

**S6 Table. Diatom taxa identified in 2014 study.** The affinity and lifeform is given after each taxon (Affinitiy: P - Polyhalobous [marine], M – Mesohalobous [marine-brackish], OH - Oligohalobion halophilous [brackish-fresh], OI - Oligohalobion indifferent [fresh-brackish], H – Halophobous [fresh]; Lifeform, the second (or third) letter: P - Planktonic/tychoplanktonic, E – Epiphytic [inc. species classified as epontic], B – Benthic).
